# Depressive Symptoms, Co-Morbidities, and Glycemic Control in Hong Kong Chinese Elderly Patients With Type 2 Diabetes Mellitus

**DOI:** 10.3389/fendo.2018.00261

**Published:** 2018-05-29

**Authors:** Annie C. H. Fung, Gary Tse, Hiu Lam Cheng, Eric S. H. Lau, Andrea Luk, Risa Ozaki, Tammy T. Y. So, Rebecca Y. M. Wong, Joshua Tsoh, Elaine Chow, Yun Kwok Wing, Juliana C. N. Chan, Alice P. S. Kong

**Affiliations:** ^1^Department of Medicine and Therapeutics, The Chinese University of Hong Kong, Shatin, Hong Kong; ^2^Li Ka Shing Institute of Health Sciences, The Chinese University of Hong Kong, Shatin, Hong Kong; ^3^School of Public Health and Primary Care, The Chinese University of Hong Kong, Shatin, Hong Kong; ^4^Hong Kong Institute of Diabetes and Obesity, The Chinese University of Hong Kong, Shatin, Hong Kong; ^5^Department of Psychiatry, The Chinese University of Hong Kong, Shatin, Hong Kong

**Keywords:** diabetes, depression, elderly, GDS-15, hypoglycaemia, comorbidities

## Abstract

**Background and objectives:**

Undiagnosed depression is an important comorbidity in type 2 diabetes (T2D) which can be detected using the Geriatric Depression Scale (GDS-15) questionnaire. In this cross-sectional study, we examined the associations of depression using GDS score with control of cardiometabolic risk factors and health status in elderly patients with T2D.

**Setting and participants:**

Between February and December 2013, patients aged ≥65 years who underwent structured comprehensive assessment as a quality improvement program at the Diabetes Center of a teaching hospital were invited to complete the GDS-15 questionnaire.

**Main outcome measures:**

Depression was defined as a GDS score ≥7. Demographic data, prior history of co-morbidities, frequency of self-reported hypoglycemia, and attainment of treatment targets defined as HbA_1c_, <7%, blood pressure <130/80 mmHg, and LDL-C <2.6 mmol/L were documented.

**Results:**

Among 325 participants (65% male, median [interquartile range] age: 69 [8] years), 42 (13%) had depression. Patients with depression had longer disease durations (mean ± SD: 15.1 ± 9.1 vs. 11.6 ± 8.1 years, *P* = 0.02), more frequent self-reported hypoglycemic events (17 vs. 6%, *P* = 0.03) and were less likely to attain all three treatment targets (0 vs. 16%, *P* = 0.004) than those without depression. On multivariable analysis, patients with depression had an odds ratio of 2.84 (95% confidence intervals: 1.35–6.00, *P* = 0.006) of reporting prior history of co-morbidities.

**Conclusion:**

In elderly patients with T2D, depression was not uncommon especially in those with poor control of risk factors, hypoglycemia, and co-morbidities. Inclusion of GDS-15 questionnaire during structured assessment for complications and risk factors can identify these high-risk patients for more holistic management of their physical and mental health.

## Introduction

Depression is an important but frequently undiagnosed comorbidity in type 2 diabetes (T2D). As a result, the impacts of mental health on self-management and quality of care are often overlooked. A previous meta-analysis reported higher odds of depression (odds ratio: 1.24–2.90) in subjects with T2D than those without T2D (1, 2), while another meta-analysis demonstrated increased risk of depression in people with diabetic complications, including retinopathy, nephropathy, macrovascular, and sexual dysfunction (3).

Depression and diabetes are complex conditions with multi-factorial etiologies. The co-existence of depression and diabetes has been associated with poor self-management and twofold increased risk of cardiovascular events, notably stroke, and premature mortality (4, 5). In Chinese adults with T2D, depression was also associated with hypoglycemia and hyperglycemia, the latter being attenuated after adjusting for suboptimal treatment adherence (6). Age is a common risk factor for both depression and diabetes (7). Thus, with increasing life expectancies, these two conditions represent major burdens on the healthcare system. Given the age-sensitive nature of depression, the Geriatric Depression Scale (GDS-15) is a screening tool that has been tailored and validated for use in the elderly population. In this study, we conducted a cross-sectional analysis from a Diabetes Register to test the utility of GDS-15 and its associations with health status and control of cardiometabolic risk factors in Chinese elderly patients with T2D.

## Materials and Methods

### Study Population and Setting

Since 1995, the Prince of Wales Hospital, the teaching hospital of the Chinese University of Hong Kong, initiated a research-driven quality improvement program, where 30–50 patients with diabetes underwent periodic structured comprehensive assessment of risk factors and complications to form the Hong Kong Diabetes Register (8). Using a unique identifier and hospitalization data, a series of risk equations were subsequently derived and validated. In 2007, together with other risk parameters, these risk scores were incorporated into the web-based Joint Asia Diabetes Evaluation (JADE) Technology for data integration, risk stratification, and issue of a personalized report to inform patients and care providers for promoting self management and individualized treatment (9). The JADE Portal was subsequently used to establish a JADE Register in Asia to improve quality of care and promote collaborative research (10).

Between February and December 2013, elderly patients aged 65 years or above diagnosed with T2D were invited to complete GDS-15 for screening of depression. The exclusion criteria included: (1) non-Chinese descent, (2) age less than 65 years old, (3) diagnosis of type 1 diabetes (acute presentation with ketosis or continuous requirement of insulin within 1 year of diagnosis of diabetes), (4) inability or refusal to provide informed consent, or (5) incomplete GDS-15 or clinical data. Ethical approval was obtained from the Chinese University of Hong Kong Clinical Research Ethics Committee with informed written consent from all participants.

### Clinical Assessment

All data were collected during a 4-h structured assessment performed by trained nurses and health care assistants after an overnight fast of at least 8 h using published protocol. These included history taking, clinical assessment, eye and feet examination, and blood and urine collection (6). Clinical data included current age and age of diagnosis for calculation of disease duration, social habits, education, family history, and frequency of self-reported hypoglycemia in the last 3 months. History of co-morbidities included history of coronary artery disease, stroke, peripheral vascular disease, chronic kidney disease, and malignancy with validation by doctors or cross-checking with the Clinical Management System. Treatment targets were adapted from the American Diabetes Association recommendations (11) and defined as HbA_1c_ <7.0%, blood pressure <130/80 mmHg, and LDL-C <2.6 mmol/L.

Depression was assessed using the Traditional Chinese version of the 15-item GDS, which has been validated in outpatient clinic settings among Hong Kong older population ≥60 years with chronic disease (12). The items involved dichotomous “yes/no” answers that make the GDS-15 easier for elderly to complete, and for scoring by clinicians administering the questionnaires. A cutoff of 7 out of 15 was defined as clinically significant depression (13, 14).

### Statistical Analysis

Data analysis was performed using the Statistical Package for Social Science software (SPSS Inc., Version 23, Chicago, IL, USA). Continuous variables were expressed as mean ± SD or median (interquartile range, IQR) and categorical variables were expressed in number (percentage). Student’s *t*-test, Chi-squared test, and Mann–Whitney *U*-test were used for group comparisons. Linear and logistic regression was performed to adjust for independent variables significantly associated with depression on univariable analysis with reporting of odds ratio (OR) and 95% confidence intervals (CI). A *P*-value <0.05 (two-tailed) was considered as statistically significant.

## Results

Between February and December 2013, 405 elderly patients with T2D provided informed consent. Of these, 53 patients who did not complete the GDS-15 questionnaire and 27 patients with incomplete clinical data were excluded from the analysis. In the remaining 325 eligible patients, 65% were male (*n* = 211) with a median age of 69 years (IQR = 8 years). The mean duration of diabetes was 12.0 ± 8.3 years and the mean HbA_1c_ was 7.2 ± 1.1%.

Table 1 compares the demographic and clinical characteristics of the study population categorized by depression defined by a validated GDS-15 score ≥7. The mean GDS-15 score of the study cohort was 3.26 ± 3.12 (range 0–15) with 13% (*n* = 42) of them reporting significant depressive symptoms based on the GDS-15 score. The depressed group had a mean GDS score of 9.71 ± 0.28 compared to 2.31 ± 1.81 in the non-depressed group. Both groups had similar distribution in term of gender, use of tobacco, and alcohol as well as use of medications for control of blood glucose, blood lipids, and blood pressure. Patients with depression had longer disease duration (15.1 ± 9.1 vs. 11.6 ± 8.1 years, *P* = 0.02) and tended to be more obese, reaching significance for waist circumference among the males (97.9 ± 8.5 vs. 91.9 ± 8.7 cm, *P* = 0.001). The depression group tended to have higher fasting plasma glucose and HbA_1c_ levels, albeit short of significance and were more likely to report hypoglycemic events within the past 3 months than the non-depressed group (17 vs. 6%, *P* = 0.030). Patients with depression had higher systolic blood pressure (145 ± 17 vs. 137 ± 19 mmHg, *P* = 0.01) with similar control in diastolic blood pressure and blood lipid values. Patients with depression were also more likely to have a prior history of diabetes-related comorbidities. When analyzed by individual events, there was a tendency for patients with depression to report a history of cardiovascular disease more frequently than the non-depression group, albeit short of significance (36 vs. 28%, *P* = 0.28).

**Table 1:** Demographic and clinical characteristics of Chinese elderly patients with type 2 diabetes with or without depression defined by Geriatric Depression Score (GDS score ≥7).

	Total	Non-depressed	Depressed	*P-*value
Number of subjects	325 (100%)	283 (87%)	42 (13%)	–
**Demographic and lifestyle details**
Age (years)	69 (61–77)	69 (62–76)	69 (60–78)	0.679
Male gender	211 (65%)	183 (65%)	28 (67%)	0.800
Duration of diabetes (years)	12.0 ± 8.3	11.6 ± 8.1	15.1 ± 9.1	0.020*
Ex or current smoker	105 (32%)	88 (31%)	17 (40%)	0.225
Ex or current alcohol drinker	133 (41%)	114 (40%)	19 (45%)	0.542
GDS-15 score	3.26 ± 3.12	2.31 ± 1.81	9.71 ± 2.28	–
**Metabolic control**
Body mass index (kg/m^2^)	25.2 ± 3.3	25.1 ± 3.2	25.7 ± 4.0	0.305
Waist circumference, men (cm)	92.7 ± 8.9	91.9 ± 8.7	97.9 ± 8.5	0.001*
Waist circumference, women (cm)	87.2 ± 8.8	87.6 ± 8.5	84.0 ± 10.1	0.160
Fasting plasma glucose (mmol/L)	7.0 ± 2.0	6.9 ± 1.9	7.5 ± 2.7	0.151
HbA_1c_ (%)	7.2 ± 1.1	7.2 ± 1.1	7.5 ± 1.0	0.081
Total cholesterol (mmol/L)	4.22 ± 0.81	4.20 ± 0.83	4.35 ± 0.64	0.277
HDL-cholesterol (mmol/L)	1.36 ± 0.38	1.36 ± 0.36	1.41 ± 0.46	0.386
LDL-cholesterol (mmol/L)	2.17 ± 0.73	2.12 ± 0.73	2.24 ± 0.69	0.514
Triglyceride (mmol/L)	1.20 (0.50–1.90)	1.20 (0.50–1.90)	1.20 (0.52–1.88)	0.565
Systolic blood pressure (mmHg)	138 ± 18	137 ± 19	145 ± 17	0.014*
Diastolic blood pressure (mmHg)	76 ± 9	75 ± 9	77 ± 9	0.382
Frequent hypoglycemic episodes (≥1 event/month)	25 (8%)	18 (6%)	7 (17%)	0.030*
**Comorbidities and medication**
History of cardiovascular events^a^	93 (29%)	78 (28%)	15 (36%)	0.275
History of any co-morbidities^b^	168 (52%)	137 (49%)	31 (74%)	0.002*
**Anti-diabetic treatment**			0.055
(1) Lifestyle only	23 (7%)	21 (7%)	2 (5%)	–
(2) On oral anti-diabetic drugs	222 (69%)	199 (71%)	23 (55%)	–
(3) On insulin	14 (4%)	12 (4%)	2 (5%)	–
(4) On both oral anti-diabetic drugs and insulin	65 (20%)	50 (18%)	15 (36%)	–
On lipid-lowering drugs	229 (71%)	197 (70%)	32 (76%)	0.383
On anti-hypertensive drugs	269 (83%)	230 (81%)	39 (93%)	0.064
**Treatment target attainment**
HbA1c <7.0%	115 (48%)	144 (51%)	11 (26%)	0.003*
LDL-cholesterol <2.6 mmol/L	241 (75%)	212 (75%)	29 (69%)	0.374
Blood pressure <130/80 mmHg	107 (33%)	101 (36%)	6 (14%)	0.006*
**Number of targets achieved**
No target attained	39 (12%)	29 (10%)	10 (24%)	–
1 Target attained	113 (35%)	95 (34%)	18 (43%)	–-
2 Targets attained	129 (40%)	115 (41%)	14 (33%)	–-
All three targets attained	44 (13%)	44 (16%)	0 (0%)	0.004*

*Data presented in mean ± SD, median (interquartile range, IQR) or number (%) (missing data excluded using exclude cases pairwise on SPSS)*.

*denotes P-value < 0.05.

*^a^Cardiovascular events include: coronary heart disease, stroke, and peripheral vascular disease*.

*^b^Any comorbidities including cardiovascular disease, chronic kidney disease (defined as estimated glomerular filtration rate less than 60 ml/min/1.73 m^2^), and any form of cancer*.

Patients with depression were less likely to achieve individual treatment target [HbA_1c_ <7.0% (26 vs. 51%, *P* = 0.003); blood pressure <130/80 mmHg (14 vs. 36%, *P* = 0.006); and LDL-C <2.6 mmol/L (69 vs. 75%, *P* = 0.37)]. Logistic regression analysis showed that depression was associated with a lower OR of attainment for blood pressure (OR: 0.33, 95% CI: 0.13–0.82, *P* = 0.017) and HbA_1c_ targets (OR: 0.35, 95% CI: 0.17–0.72, *P* = 0.005) and higher odds for having a prior history of diabetes-related complications (OR = 2.85, 95% CI: 1.35–6.00, *P* = 0.006) after adjusting for age, gender, and disease duration (Table 2). None of the patients with depression attained all three treatment targets of HbA_1c_, LDL-C, and blood pressure versus those without depression (0 vs. 16%, *P* = 0.004). On linear regression analysis, after adjustment for confounders, depressed subjects had a lower OR for attaining all three treatment targets than those without depression (Table 3).

**Table 2:** Logistic regression analysis for risk association between depression (GDS score ≥7) and control of cardiometabolic risk factors and co-morbidities in Chinese elderly patients with type 2 diabetes.

		Odds ratio (95% confidence interval)	*P*-value
Blood pressure <130/80 mmHg target attainment	Model 1	0.330 (0.133–0.823)	0.017*
	Model 2	0.323 (0.127–0.819)	0.017*
	Model 3	0.301 (0.121–0.748)	0.010*
	Model 4	0.292 (0.118–0.726)	0.008*
HbA_1c_ <7.0% target attainment	Model 1	0.349 (0.168–0.724)	0.005*
	Model 2	0.441 (0.201–0.968)	0.041*
	Model 3	0.409 (0.188–0.890)	0.024*
	Model 4	0.400 (0.188–0.853)	0.018*
History of any co-morbidities^a^	Model 1	2.844 (1.347–6.002)	0.006*
	Model 2	2.559 (1.178–5.562)	0.018*
	Model 3	2.850 (1.349–6.021)	0.006*
	Model 4	2.853 (1.354–6.013)	0.006*

*Model 1: Adjusted for age, gender and use of anti-hypertensive drugs*.

*Model 2: Adjusted for age, gender, use of anti-diabetic drugs, insulin, lipid lowering drugs and anti-hypertensive drugs*.

*Model 3: Adjusted for age, gender, use of anti-diabetic drugs, and use of insulin*.

*Model 4: adjusted for age, gender, and duration of diabetes*.

*denotes P-value < 0.05.

*^a^Any comorbidities including cardiovascular, chronic kidney disease, and any form of cancer*.

**Table 3:** Linear regression analysis for risk association of depression (GDS score ≥7) with control of cardiometabolic risk factors in Chinese elderly patients with type 2 diabetes.

		Regression coefficient (95% confidence interval)	*P*-value
Systolic blood pressure	Model 1	6.425 (0.652–12.197)	0.029*
	Model 2	6.678 (0.853–12.504)	0.025*
All three targets attained	Model 1	−0.500 (−0.780 to −0.219)	0.001*
	Model 2	−0.439 (−0.716 to −0.162)	0.002*

*Model 1: adjusted for age, gender, and use of anti-hypertensive drugs*.

*Model 2: adjusted for age, gender, duration of diabetes, use of anti-diabetic drugs, insulin, lipid lowering drugs, and anti-hypertensive drugs*.

*denotes P-value < 0.05.

## Discussion

In this cross-sectional analysis of Chinese elderly patients with T2D, 13% of them had depression which was associated with longer disease durations, more frequent self-reported hypoglycemic events, reduced likelihood of attaining all three treatment targets, and increased odds of having co-morbidities compared to those without depression.

### The Bidirectional Association Between Depression and Diabetes

Depression and diabetes exhibit bidirectional relationships (7) and share pathophysiological mechanisms, such as increased oxidative stress, low grade inflammation, and hypercortisolemia (15–17). Depression has been suggested as a risk factor for diabetes for several reasons. First, impaired mental health and lack of motivation might worsen medication adherence and obesity due to suboptimal lifestyle factors, such as physical inactivity and smoking (18). Second, increased activation of the hypothalamic–pituitary–adrenal axis, sympathetic nervous and immune systems in response to stress could lead to hypercortisolemia, increased gluconeogenesis, and reduced insulin secretion (7).

On the other hand, patients with diabetes are prone to develop depression. The emotional burden of a diabetes diagnosis and possible development of complications can lead to rumination and failure to concentrate, which may result in memory, cognitive, and functional impairment. In elderly patients, dependency of daily activities may result in poor self-perception of health, social withdrawal, fear of falling, sense of loneliness, and reflection of past life events which may worsen depressive symptoms. These negative moods can be further aggravated by the co-occurrence of diabetes (19–21).

### Depression in the Elderly

In elderly, emotional and somatic symptoms of depression may be concealed by or mistaken for cognitive impairment or dementia making an accurate diagnosis of depression challenging. Yet, even in primary care settings, depression is treatable using psychoeducation, psychotherapy, and medications, followed by monitoring and referral when necessary. There is also evidence suggesting that relief from depression could improve glycemic control (1), but the converse has not been reported. In a randomized controlled trial conducted in primary care setting, integrated care of diabetes and depression in adults improved adherence to oral antidiabetic medications and antidepressants with improved glycemic control and reduced depressive symptoms compared to usual care (22). In “Diabetes, depression, and death: a randomized controlled trial of a depression treatment program for older adults based in primary care” (PROSPECT), depressed elderly patients with diabetes who received intervention were less likely to die during a 5-year period compared to the usual care group with an adjusted hazard ratio of 0.49 (95% CI [0.24–0.98]) (23).

The GDS-15 is a shortened version of GDS-30 and can be self-administered. It is widely accepted by elderly patients and healthcare professionals for its brevity, simplicity, and sensitivity with dichotomous items of “yes or no.” Previous studies using GDS-15 in Hong Kong Chinese population mainly focused on patients with stroke or suicide ideation (24). Our results confirmed the utility of GDS-15 in identifying patients with diabetes and comorbid depression which was associated with complications and poor control of cardiometabolic risk factors.

In our study cohort, 13% of patients had depression. Earlier studies conducted in Hong Kong Chinese reported higher prevalence of depression in elderly with T2D (12.2%) than those without diabetes (9%) (25). In most surveys, depression is more common in women than men. An earlier study in Hong Kong also reported a female preponderance of depression in elderly population (14.5 vs. 11%) (26). Possible reasons for this gender difference included higher levels of psychological stress, somatic complaints, and poor self-rated health in females (27). However, we did not find this gender difference which might be due to the male predominance and small sample size of our study cohort.

### Depression Is Associated With Co-Morbidities and Poor Control of Cardiometabolic Risk Factors

In agreement with the literature (28), Chinese elderly patients with T2D were more likely to report prior history of co-morbidities. Although we did not measure treatment adherence in this study, we have reported its association with depression using PHQ9 in a separate cohort with broader age range (6). In a review article, the authors highlighted that comorbid depression might increase disease burden of T2D due to poor self-management, increased risk of dementia and healthcare expenditure (28). Besides, aging and diabetes are both linked to dependency, psychosocial, and physical changes, which can be exacerbated under the synergistic effect of depression (29). Functional disability, defined as difficulty performing routine activities of daily living or social activities, can be both a consequence of and a risk factor for developing chronic diseases. In this light, patients with comorbid depression and diabetes were more likely to experience functional disability than those with either diabetes or depression alone (30). In our analysis, elderly patients with diabetes and depression were more likely to have co-morbidities, including cardiovascular disease, chronic kidney disease, and cancer, which was likely to result in functional disability although this was not formally documented.

In agreement with our previous report (31), elderly patients with depression had higher HbA1c and frequency of hypoglycemic episodes than those without depression. This combined trait of hyperglycemia and hypoglycemia can lead to glycemic variability which has been shown to predict depressive symptoms in Israeli elderly patients with T2D (32). In the same study, the association was observed in patients irrespective of attainment of HbA_1c_ target <7% although the effect size was more evident in those who were not at target. In a recent prospective analysis of the Hong Kong Diabetes Register, we have reported a hazard ratio of 1.5 of cardiovascular-renal disease for every 1 SD in HbA1c (33). In this cross-sectional study, we only used the HbA1c value and self-reported hypoglycemic events in the past 3 months collected during the comprehensive assessment for analysis. Thus we were not able to examine the association between depression and HbA1c variability which requires prospective evaluation.

Apart from poor glycemic control, none of our elderly patients with diabetes and depression had attained any of the three treatment targets compared to 16% in those without depression. Attainment of these treatment targets had prognostic significance for future clinical outcomes. In this light, we have reported that patients who attained two or more treatment targets had 50% lower risk of incident cardiovascular disease than who were not at any target (34). These constellations of poor control of cardiometabolic risk factors, frequent hypoglycemia, co-morbidities, poor self management, and functional disability might contribute to the 1.5-fold increased risk of mortality in patients with diabetes and depression compared to diabetes alone (35), thus highlighting the importance in identifying these high risk patients for holistic management.

### Social Support and Multidisciplinary Care in Elderly Patients With Diabetes and Depression

Achieving optimal glycemic control is more challenging in elderly than younger patients for many reasons. Age-related functional, cognitive, and physical impairments can hinder an individual’s ability to remember and comply with complex self-management regimens including changes to their accustomed lifestyles (25). A holistic approach addressing the biopsychosocial needs in elderly patients with comorbid depression could improve quality of care and reduce the burden on their family members and healthcare services. In this light, satisfaction of elderly patients toward family and social support has been shown to protect against development of depressive symptoms (36, 37).

In Hong Kong, since 2000, periodic comprehensive assessment for risk factors and complications performed by trained nurses is a standard service in the public healthcare institutions in both hospital and primary care settings (38). Our results suggested that inclusion of GDS-15, especially in those with poor control of risk factors and/or co-morbidities might identify elderly patients with depression, who would benefit from intensive health and social care support by a multi-disciplinary team, including endocrinologists, primary care doctors, and psychiatrists and other professional and community care workers to address these multiple needs.

### Study Limitations

Several limitations of this study should be noted. First, the study cohort was relatively small, and all patients were recruited from a single hospital. Second, screening of depression was based solely upon GDS score without confirmation using standard instruments such as the Mini International Neuropsychiatric Interview or clinical diagnosis by a psychiatrist. While we were unable to comment on the diagnostic performance of GDS-15 for depression, the tool was useful in identifying a subgroup of patients with unmet needs. Third, we did not document the personal or family history of depression or administration of any psychotropic medications. The latter could be retrieved from the Clinical Management System of the hospital but this was outside the scope of this study. Besides, many of these electronic medical records were used to collect data and not designed to establish a register for quality improvement or monitoring purpose, making data-mining, data-cleaning, and data-analysis extremely labor-intensive. Our experience of redesigning the workflow for systematic collection of data periodically to establish a Register could combine research and practice to address multiple purposes including quality assurance, providing feedback to care providers, and patients with ongoing evaluation to identify unmet needs (9). Finally, these cross-sectional data are only hypothesis-generating. Longitudinal and interventional studies are needed to confirm whether alleviation of depression could improve risk factor control and clinical outcomes in elderly patients with T2D and depression.

### Implications for Clinical Practice

In elderly Chinese people with T2D, 13% had depression which was associated with long disease duration, poor control of cardiometabolic risk factors, frequent hypoglycemia and co-morbidities. Patients harboring these traits would benefit for screening for depression using GDS-15. Based on prior work, these patients had high risk for functional disability and death. Thus, upon identification, these patients would require detailed cognitive, psychological, and behavioral assessments followed by holistic health and social care in order to improve psychological health, self management, drug adherence, control of cardiometabolic risk factors, and clinical outcome.

## Conclusion

In hospital clinic setting, depression affects 13% of Chinese elderly patients with T2D. Patients with long disease duration, poor control of cardiometabolic risk factors, frequent hypoglycemia and comorbidities are high risk individuals for having comorbid depression. Given the silent nature of these risk factors and depression, periodic comprehensive assessment of risk factors, and complications including the use of GDS-15 may identify high risk patients for early intervention in order to reduce the disease burden on patients, their families, and healthcare services.

## Ethics Statement

This study received approval from the Chinese University of Hong Kong (CUHK) Clinical Research Ethics Committee and informed written consent was obtained from all participants.

## Author Contributions

AF: data collection, analysis, manuscript drafting, and critical revision. GT: data interpretation, analysis, and manuscript critical revision. HC and EL: data collection, interpretation, and analysis. AL, RO, TS, RW, JT, and EC: data interpretation, manuscript critical revision. YW, JC, and AK: data interpretation, manuscript critical revision, and project supervision.

## Conflict of Interest Statement

The authors declare that the research was conducted in the absence of any commercial or financial relationships that could be construed as a potential conflict of interest.
